# A Case of Nephrotic Syndrome, Showing Evidence of Response to Saquinavir

**DOI:** 10.1155/2015/512549

**Published:** 2015-01-31

**Authors:** Giles Walters, Faisal A. Choudhury, Budhima Nanayakkara

**Affiliations:** ^1^Department of Renal Medicine, Canberra Hospital, Garran, ACT 2605, Australia; ^2^Australian National University Medical School, Canberra, Australia; ^3^The Canberra Hospital, Australia

## Abstract

The treatment of primary nephrotic syndrome such as minimal change nephropathy, membranous nephropathy, and focal segmental glomerulosclerosis nephropathy remains challenging. Whilst most cases of idiopathic nephrotic syndrome respond to steroid therapy and experience a limited number of relapses prior to complete remission, some cases suffer from frequent relapses and become steroid dependent or are primarily steroid resistant. Treatment options are limited to immunosuppressive drugs with significant side effect profiles. New modalities targeting novel pathways in the pathogenesis of nephrotic syndrome are actively sought. Here we report the case of a patient with steroid dependent focal segmental glomerulosclerosis (FSGS) nephrotic syndrome with a favourable response to a novel proteasome inhibitor saquinavir.

## 1. Introduction

This case study highlights the potential use of saquinavir, a proteasome inhibitor, to treat a difficult case of steroid dependent nephrotic syndrome. Saquinavir as long-term therapy is well tolerated amongst seropositive HIV patients [1, 2] and is known to reduce HIV induced proteinuria [3]. In addition to its HIV protease inhibition, it also acts to inhibit the proteasome, a key factor in the regulation of NF-*κ*B activity [4–6]. The proteasome was implicated in pathogenesis by studies showing that NF-*κ*B is elevated in circulating peripheral blood mononuclear cells in patients with idiopathic nephrotic syndrome [7, 8]. This was confirmed by T-cell transcriptome analysis [9]. Elevation of NF-kappa beta in T cells and its subsequent translocation into the nucleus lead to synthesis of circulating T-cell factors causing podocyte cytoskeleton disorganisation, increased glomerular permeability, and eventual proteinuria [10].

## 2. Case Study: Nephrotic Syndrome, Showing Evidence of Response to Saquinavir

The patient is a 23-year-old male of African Caucasian background first diagnosed with nephrotic syndrome at the age of 4. He has been on multiple immunosuppressive regimes including cyclophosphamide, levamisole, rituximab, cyclosporine, tacrolimus, and mycophenolate mofetil. During his lifetime he has been a frequent relapser showing poor response to many steroid sparing medications. At his latest admission for relapse of nephrotic syndrome (biopsy confirmed FSGS) he was given a trial of antiretroviral proteasome inhibitor saquinavir and prednisolone followed by maintenance with saquinavir, prednisolone, and low dose cyclosporine. We report an initial response to this regime, adding support to the small number of existing cases regarding the therapeutic efficacy of saquinavir in the treatment of nephrotic syndrome.

This patient was initially seen at the Department of Renal Medicine at the Canberra Hospital in 2009 as an 18-year-old male, who otherwise had no major medical or surgical comorbidities. He had his first episode of nephrotic syndrome at the age of 4 in another centre, and since then he had been a frequent relapser but steroid responsive. Prior to being seen at our clinic, he had received two courses of cyclophosphamide in 1995 (2 mg/kg for 72 days and then 1.7 mg/kg for 90 days). Initial biopsy in 1995 revealed only minimal change. Levamisole was started as a steroid sparing agent, but after 4 years he developed a serum-sickness-like syndrome. Subsequently, levamisole was switched to cyclosporine. He was maintained on therapeutic cyclosporine for 2 years. A repeat biopsy showed prominent peritubular calcification consistent with calcineurin inhibitor toxicity. A decision to decrease cyclosporine was made and in early 2007 he had a further relapse with resistant nephrotic syndrome despite methylprednisolone and high dose diuretics. His admission was complicated by hypertension and pulmonary oedema. A biopsy at admission showed FSGS. Following his discharge from hospital his oedema and hypoalbuminaemia persisted. A decision was made to initiate rituximab therapy and he was given 3 doses in October 2007. Despite the depletion of his CD19 cells there was no remission in his clinical symptoms and he remained oedematous. He was restarted on cyclosporine and high dose prednisolone achieving remission. From this time onwards he was reviewed in clinic at the Canberra Hospital, with nearly monthly reviews following his renal function, fluid status, and blood pressure. A further relapse occurred in April 2010, after which cyclosporine was switched to tacrolimus. This resulted in remission, and the patient was able to taper his prednisolone to doses of 5 mg daily. A repeat biopsy in 2010 showed FSGS. Unfortunately from 2010 until 2012 this patient had 2 further relapses, and in-between relapses could not be weaned off prednisolone. Monthly clinical reviews revealed persistent hypoalbuminaemia, proteinuria, raised creatinine trending above baseline, and hypertension. Mycophenolate mofetil (1 g BD) was considered as a possibility given the already exhaustive list of immunosuppressive medications trialled with limited effect. Initiation of this purine synthesis inhibitor in addition to tacrolimus leads to normalisation of creatinine and serum albumin levels. During this trial period he had an episode of acute renal impairment with creatinine rising to 220 micromol/L and albumin falling to 25 mg/dL. Additionally the patient complained of intolerable gastrointestinal side effects with worsening hypertension. Mycophenolate mofetil was ceased, which unfortunately led to acute renal impairment in December 2012 with creatinine levels rising to 267, albumin of 21, and increased tissue oedema. He was admitted for in-patient management where tacrolimus was ceased and hypervolemia was treated with aggressive frusemide therapy and fluid restriction. A trial of saquinavir 1 g BD with prednisolone 75 mg daily was started. Approximately one month following discharge from hospital, this patient's oedema had fully resolved and creatinine resolved to near baseline levels. Prednisolone was gradually weaned to 15 mg daily with the introduction of low dose cyclosporine (10 mg BD), much lower than previous doses used in maintaining remission. The patient remained in remission for 2 months. Unfortunately in May 2013 he had a further relapse into nephrotic syndrome and high dose prednisolone (75 mg daily) was required for remission. He is currently maintained in a state of remission with the following medications and doses: prednisolone 5 mg daily, saquinavir 1 gm BD, and cyclosporine 10 mg BD. Apart from his steroid dependent nephrotic syndrome, his only other medical comorbidity is gout which is well controlled on allopurinol 100 mg daily. He remains hypertensive requiring multiple antihypertensive medications including ramipril, minoxidil, and atenolol.

## 3. Discussion

The combination of saquinavir with high dose prednisolone as induction therapy, followed by a maintenance regime of saquinavir, low dose prednisolone, and low dose cyclosporine, allowed for a period of remission in this patient, which was unfortunately interrupted with a brief relapse. We speculate that saquinavir's unique mechanism of action on proteasome, in combination with glucocorticoid and calcineurin inhibitor, promotes the improvement and stabilisation of biochemical parameters and control of clinical symptoms by downstream inhibition of NF-*κ*B changes.

Indeed, the proteasome is a vital component in controlling NF-*κ*B nuclear translocation and therefore transcriptional regulation [4]. Following activation of NF-*κ*B by cytokines such as TNF-alpha and IL-1, I-kappa-beta, the inhibitory regulator of NF-*κ*B that keeps this protein localised to the cytoplasm, is first phosphorylated then ubiquitinated [4]. Ubiquitination targets I-kappa-b for proteosomal degradation, allowing the nuclear localisation sequence of NF-kappa beta to be unveiled [4]. Addition of saquinavir to peripheral blood mononuclear cells isolated from patients with idiopathic nephrotic syndrome has been shown to reduce the nuclear fraction of NF-kappa beta [10]. Coppo et al. (2012) [10] were the first to trial saquinavir as therapy for difficult cases of idiopathic nephrotic syndrome in children and young adults. Coppo et al. (2012) used saquinavir in addition to other immunosuppressive medications to treat 10 patients with idiopathic nephrotic syndrome. Eight patients continued the treatment for between 12 and 68 months and six of these patients responded to this agent [10]. In addition, there was a 63% reduction in prednisone use associated with a significant reduction in steroid toxicity [10]. This study also reported that saquinavir was effective when used with calcineurin inhibitors at doses much lower than required to induce remission when used without saquinavir [10]. Furthermore, given that therapeutic levels of cyclosporine inhibitors were within the therapeutic range during treatment (as was true in this case), it is unlikely that remission induced by the saquinavir/cyclosporine regime was solely attributable to pharmacokinetic modification of calcineurin inhibitors by saquinavir [11]. Remission induced in this patient following the latest relapse occurred with combination of glucocorticoid, calcineurin inhibitor, and saquinavir. Glucocorticoids act on glucocorticoid receptors promoting nuclear translocation, binding to NF-*κ*B, and repressing its function [12, 13]. Furthermore, cyclosporine A has been shown to be a noncompetitive inhibitor of the proteasome [14], with* in vivo* suppression of lipopolysaccharide induced I-*κ*B degradation [14]. Therefore, it is tempting to speculate that the combined suppressive effects of cyclosporine, prednisolone, and saquinavir on NF-kappa beta nuclear translocation are, at least in part, responsible for reduction in proteinuria and induction of remission in our patient [10].

## 4. Conclusion

The case demonstrates that the introduction of saquinavir and high dose prednisolone followed by maintenance therapy with saquinavir and low dose prednisolone and cyclosporine has the potential to induce and perhaps maintain remission in patients with difficult-to-treat steroid responsive nephrotic syndrome. Care must be taken, however, in recommending the addition of saquinavir to the treatment regime of such patients. The numbers of absolute cases treated with saquinavir that have been published remain small. Randomised control trials with greater power are required in order to clarify whether treatment with saquinavir will reach statistical significance in reducing relapse frequency and decreasing steroid use/dependence in this difficult-to-treat condition. This case study adds to the growing evidence showing that saquinavir may be useful in inducing remission by decreasing proteinuria and hypoalbuminuria in patients with steroid dependent or steroid resistant idiopathic nephrotic syndrome. We would only recommend this as rescue therapy currently or as a further step in managing difficult nephrotic patients.
